# Combining mKate2-Kv1.3 Channel and Atto488-Hongotoxin for the Studies of Peptide Pore Blockers on Living Eukaryotic Cells

**DOI:** 10.3390/toxins14120858

**Published:** 2022-12-05

**Authors:** Nikita A. Orlov, Anastasia A. Ignatova, Elena V. Kryukova, Sergey A. Yakimov, Mikhail P. Kirpichnikov, Oksana V. Nekrasova, Alexey V. Feofanov

**Affiliations:** 1 Shemyakin-Ovchinnikov Institute of Bioorganic Chemistry, Russian Academy of Sciences, 117997 Moscow, Russia; 2 Faculty of Biology, Lomonosov Moscow State University, 119234 Moscow, Russia

**Keywords:** Kv1.3 channel, pore blocker, fluorescence, confocal, patch clamp, affinity, hongotoxin, competitive binding

## Abstract

The voltage-gated potassium Kv1.3 channel is an essential component of vital cellular processes which is also involved in the pathogenesis of some autoimmune, neuroinflammatory and oncological diseases. Pore blockers of the Kv1.3 channel are considered as potential drugs and are used to study Kv1 channels’ structure and functions. Screening and study of the blockers require the assessment of their ability to bind the channel. Expanding the variety of methods used for this, we report on the development of the fluorescent competitive binding assay for measuring affinities of pore blockers to Kv1.3 at the membrane of mammalian cells. The assay constituents are hongotoxin 1 conjugated with Atto488, fluorescent mKate2-tagged Kv1.3 channel, which was designed to improve membrane expression of the channel in mammalian cells, confocal microscopy, and a special protocol of image processing. The assay is implemented in the “mix and measure”, format and allows the screening of Kv1.3 blockers, such as peptide toxins, that bind to the extracellular vestibule of the K^+^-conducting pore, and analyzing their affinity.

## 1. Introduction

The voltage-gated potassium Kv1.3 channel mediates potassium ion flux through the membranes contributes for a number of cellular processes, including propagation of action potential in neurons and muscle cells, as well as proliferation, migration, and secretion of hormones and cytokines in non-excitable cells. The Kv1.3 channel is involved in the pathogenesis of autoimmune [1], neuroinflammatory [2], and oncological diseases [3].

Pore blockers of the Kv1.3 channel, whether they are small organic molecules or polypeptide toxins, plug the ion conducting pathway and, thus, exert an inhibitory effect on the channel. Many of them are considered as potential drugs to treat pathological conditions, which are associated with either over expression or hyperactivity of the channel [4,5,6,7]. Among them, some peptide toxins of venomous origin (namely, from scorpions, sea anemones, some species of snakes, and sea snails) that bind to the external vestibule of Kv1.3 channel pore are distinguished by high affinity and selectivity to the target channel [8,9]. Besides prospective medical application, they are used to study Kv1 channels’ structure and functions [8,10,11].

Discovery, design, and production of Kv1.3 channel pore blockers are important research and pharmacological tasks, which are closely related with the assessment of their ability to bind the channel [12].

The affinity constants of the pore blockers measured with respect to their target channels are most often obtained using electrophysiological techniques, which are considered the most informative functional methods for studying ligand-channel interactions [13]. Alternative methods include radioligand analysis [14,15], Rb^+^ flux assays [16], and some fluorescence-based approaches that utilize mainly voltage-sensitive dyes or fluorescent ion indicators [17].

Fluorescently labeled peptide ligands, which directly block the Kv channel pore, can be used to develop new formats of ligand binding assays. These ligands can be produced either by chemical conjugation of the peptide blockers with fluorescent organic dyes or by fusing the target peptides with fluorescent proteins by bioengineering methods [18,19,20]. Both types of fluorescent peptides were shown to bind Kv1 channels in eukaryotic cells, revealing channel expression and distribution [21,22,23].

Moreover, fluorescent ligands can be applied similarly to radio labeled ligands to search for pore blockers among individual compounds, or in complex mixtures to study their properties. This was demonstrated in assays utilizing a fluorescent ligand and KcsA-Kv1 chimeric channels expressed in the membranes of bacteria [24]. The quantitative analysis of the competitive binding of fluorescent and non-fluorescent pore blockers to the KcsA-Kv1 channel was shown to provide a reliable estimation of the dissociation constant of the complex between the channel and a non-fluorescent pore blocker [24]. However, until now, fluorescent peptides have never been used to determine the affinity of non-fluorescent ligands to the target Kv1 channel on living mammalian cells.

To routinely use fluorescence imaging for studying Kv1.3 blockers on mammalian cells, a significant expression level of the channel should be achieved and controlled using fluorescence. Fortunately, it was demonstrated that Kv1.3 channel tagged with fluorescent proteins can be expressed in eukaryotic cells with preservation of their functional activity. Examples of such functional expression include rat Kv1.3 fused with YFP [25,26], eGFP-tagged murine or human Kv1.3 [27,28] and human Kv1.3 fused with mCherry [29].

In our preliminary report on microscopy and microanalysis meeting, the membrane presentation of human Kv1.3 tagged with red fluorescent protein mKate2 was demonstrated and its binding with hongotoxin 1 (HgTx1) labeled with Atto488 fluorophore (A-HgTx) was shown in mammalian cells [30]. In the present work, we report on the optimized design of human Kv1.3 fused with mKate2 that provides high membrane presentation of the channel in mammalian HEK293 and Neuro2a cells and preserves its physiological activity. The peculiarities of the cellular distribution of different constructions of mKate2-tagged Kv1.3 are discussed. It is shown that A-HgTx possesses high affinity to mKate2-tagged Kv1.3, blocks K^+^ currents through the channel and has negligible non-specific binding to other membrane components. Using A-HgTx and mKate2-tagged Kv1.3, a general approach to the quantitative analysis of a fluorescent ligand binding to a fluorescent ion channel on the membrane of mammalian cells was developed. The approach was demonstrated to be applicable to measure the dissociation constant of the complex between Kv1.3 channel and a fluorescent ligand and to estimate the affinities of non-fluorescent Kv1.3 ligands in the competitive binding experiments.

## 2. Results and Discussion

### 2.1. Design of mKate2-Tagged Kv1.3 Channels

To provide a basis for fluorescence microscopy studies of interactions between peptide blockers and Kv1.3 channel using mammalian cells, a high level of visualizable channel presence at the plasma membrane should be achieved. It is easier to solve this task if the channel fluoresces. Accordingly, we designed plasmids encoding the α-subunit of human Kv1.3 fused with bright red fluorescent protein mKate2 [31] at the N- or C-terminus via a short flexible polypeptide linker (K-Kv1.3 and Kv1.3-K, respectively). mKate2 was used for tagging potassium channels for the first time. 

**Figure 1:**
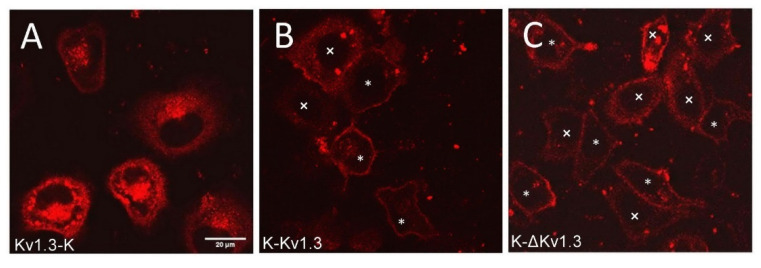
Confocal fluorescent images of distribution of Kv1.3-K (**A**), K-Kv1.3 (**B**) and K-ΔKv1.3 (**C**) channels in HEK293 cells. Scale bar—20 µm. Two types of channel distribution were observed in cells for K-Kv1.3 (**B**) and K-ΔKv1.3 (**C**): prevailing membrane presentation (cells marked with *) and dominating cytoplasmic localization (cells marked with ×).

The expression of mKate2-tagged Kv1.3 channels was observed in 30–40% of the cells subjected to transfection. Transient expression of K-Kv1.3 and Kv1.3-K channels in HEK293 cells revealed essentially different patterns of cellular distribution of the channels, indicating that the mKate2 position in the fusion protein construct affects localization of Kv1.3 in cells (Figure 1A,B). Characteristic features of Kv1.3-K distribution in HEK293 cells are prevailing web-like and granular accumulation in cytoplasm (with predominant localization of the granules near a nucleus) and relatively moderate presentation on the plasma membrane (Figure 1A). Notably, the images were selected and their description was performed after three-dimensional scanning of cells using confocal laser scanning microscopy to further ensure that the presented optical sections pass through the equatorial plane of the cell nucleus, and not through the upper or lower layers of the plasma membrane.

In the case of K-Kv1.3, two types of channel-expressing cells were observed. Many cells demonstrated dominating membrane localization of K-Kv1.3, which was accompanied by a weak diffuse distribution in the cytoplasm and by the appearance of K-Kv1.3-containing cytoplasmic granules in some cases (Figure 1B). In such cells, the number of granules with K-Kv1.3 was significantly less, while the membrane presentation of channels was more evident than in the case of Kv1.3-K (Figure 1A,B). In some cells, distribution of K-Kv1.3 was similar to that of Kv1.3-K: cytoplasmic distribution of the channel dominating over its presentation in the plasma membrane (Figure 1B).

Unlike rodent Kv1.3 channels, which are often expressed in mammalian cell lines or in *Xenopus oocytes* for electrophysiological studies, the human Kv1.3 channel is encoded by gene *KCNA3* with two alternative start ATG codons that correspond to M1 and M53 residues of the channel. Shortened transcript of *KCNA3* gene was found to encode truncated but fully functional K^+^ channel (ΔKv1.3), whose activity and the ability to bind peptide blocker charybdotoxin (ChTx) are similar to the properties of Kv1.3 in mouse and human T lymphocytes [32,33]. While membrane expression of the full-length human Kv1.3 was poor in *X. oocytes*, deletion of N-terminal M1-D52 residues boosted the ΔKv1.3 insertion into membrane, suggesting a potential regulatory role of N-terminal sequence in human Kv1.3 channel expression [34]. It has been shown that the ΔKv1.3 channel fused with eGFP or mCherry targets the plasma membrane of HEK293 and CHO cells and preserves K^+^ conductivity [35].

To test the possibility of enhancing the membrane expression of human Kv1.3 by removing its N-terminal fragment, we created a plasmid encoding N-terminally truncated Kv1.3 fused at the N-terminus with mKate2 (K-ΔKv1.3). Qualitatively, the patterns of K-ΔKv1.3 expression in HEK293 cells were found to be similar to those described above for K-Kv1.3 (Figure 1B,C). In cells that demonstrated prevailing membrane localization of the channel, quantitative analysis of fluorescence intensity revealed slightly higher membrane presentation of K-ΔKv1.3 as compared to K-Kv1.3 (Supplementary Materials, Figure S1).

### 2.2. Intracellular Localization of Channels

Features of intracellular distribution of K-ΔKv1.3, Kv1.3-K, and K-Kv1.3 were clarified using fluorescent markers of cellular organelles and laser scanning confocal microscopy.

**Figure 2:**
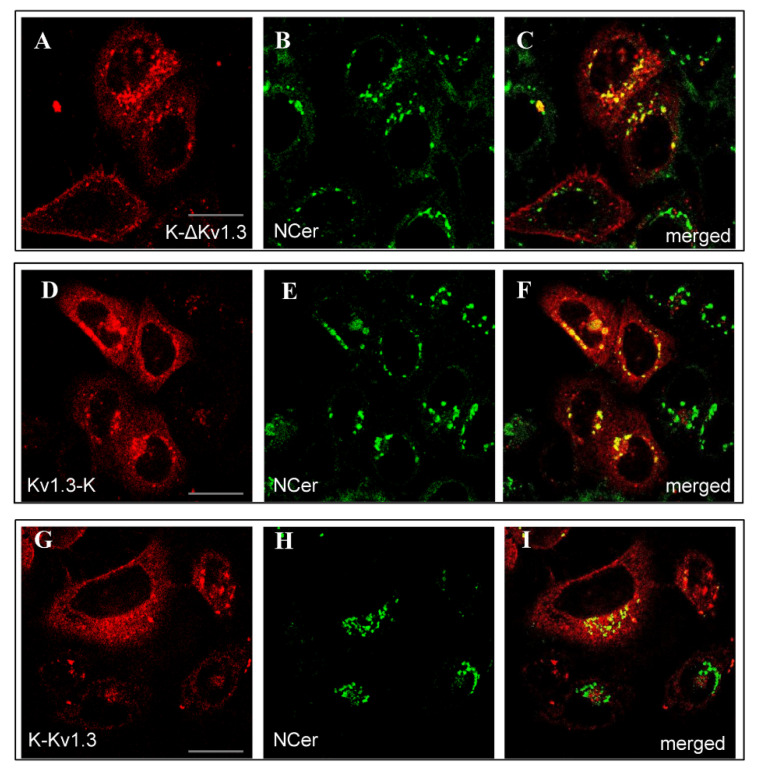
Distribution of mKate2-tagged Kv1.3 (red) and the Golgi marker NCer (green) in living HEK293 cells. (**A**–**C**) Cells expressing K-ΔKv1.3, (**D**–**F**) Cells expressing Kv1.3-K, (**G**–**I**) Cells expressing K-Kv1.3. (**C**,**F**,**I**) Merged images of mKate2-tagged Kv1.3 and NCer fluorescence. Yellow color indicates co-localization of the channels and NCer in the Golgi apparatus. Scale bar: 20 µm.

K-ΔKv1.3, Kv1.3-K, and K-Kv1.3 were found to accumulate in the Golgi apparatus as followed from co-localization of the fluorescence of NBD C6-Ceramide (NCer), marker of *trans*-Golgi apparatus, and the channel fluorescence (Figure 2). According to this study, a considerable part of cytoplasmic granules with the channels are compartments of the Golgi apparatus. This observation applies to all the cells intensely expressing Kv1.3-K channels (Figure 2D–F) and to fractions of cells with dominating cytoplasmic distribution of K-ΔKv1.3 and K-Kv1.3 (Figure 2A–C,G–I). Cells, where membrane presentation of channels prevails, showed no accumulation of K-ΔKv1.3 and K-Kv1.3 in the Golgi apparatus (Figure 2A–C,G–I). 

**Figure 3:**
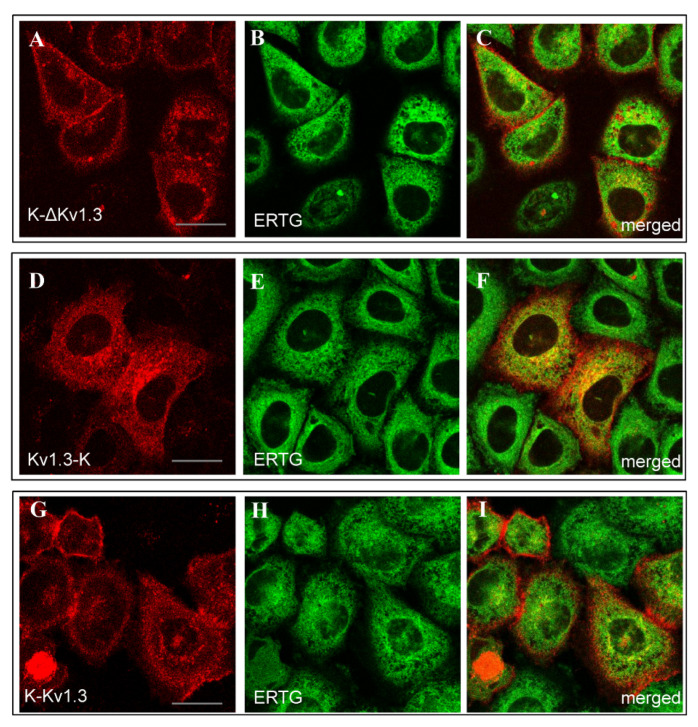
Distribution of mKate2-tagged Kv1.3 (red) and the endoplasmic reticulum marker ERTG (green) in living HEK293 cells. (**A**–**C**) Cells expressing K-ΔKv1.3. (**D**–**F**) Cells expressing Kv1.3-K. (**G**–**I**) Cells expressing K-Kv1.3. (**C**,**F**,**I**) Merged images of mKate2-tagged Kv1.3 and ERTG fluorescence. Yellow color indicates co-localization of the channels and ERTG in the endoplasmic reticulum. Scale bar: 20 µm.

The web-like distribution of Kv1.3-K was found to be similar to the distribution of ER Tracker Green (ERTG) in the endoplasmic reticulum (ER, Figure 3D–F). Diffuse cytoplasmic distributions of K-ΔKv1.3 and K-Kv1.3 showed some signatures of co-localization with ERTG, but a definite conclusion is complicated because of a weak cytoplasmic fluorescence of the channels (Figure 3A–C,G–I). 

The studies with endosome marker transferrin conjugated with Alexa Fluor 488 (TR488, Figure S2) and lysosome marker Lyso Tracker Green (LTG, Figure S3) revealed the presence of K-ΔKv1.3, Kv1.3-K, and K-Kv1.3 in some endosomes and lysosomes. 

No expression of K-ΔKv1.3, Kv1.3-K, and K-Kv1.3 in mitochondria was found using the mitochondrion marker rhodamine 123 (Rh123, Figure S4).

It is notable that the pattern of sub cellular localization of full-length Kv1.3 is characterized by a balance of membrane-embedded and cytoplasmic pools of the channel, which is related to the level of Kv1.3 signaling and thereby crucial for cell physiology [36]. Biogenesis of Kv1.3 channels begins in rough ER where the synthesis and tetramer assembly of the channels occur. The channels exit ER and undergo anterograde trafficking to ER-Golgi intermediate compartment and further through the Golgi cisternae to target the plasma membrane [36]. This channel trafficking is guided by various mechanisms, including special signal sequences and targeting motifs encoded in the structure of Kv1.3 channel. The C-terminal domain of Kv1.3, which contains two membrane-targeting acidic motifs, was found to be essential for the channel trafficking to immunological and neural synapses [28,37]. Deletion of the C-terminus of Kv1.3 resulted in a dramatic ER retention of the channel in HEK293 cells, thus, preventing their membrane presentation [28]. 

In our case, when mKate2 was attached to the C-terminus of Kv1.3, retention of the channel in the ER and Golgi apparatus was obviously enhanced as compared to Kv1.3 bearing mKate2 at the N-terminus (Figure 1). It seems that C-terminal mKate2 hampers Kv1.3 traffic from ER to plasma membrane. Similarly, considerable retention of human Kv1.3 channels in cytoplasm of HEK293 cell was observed when the channels were C-terminally tagged with either GFP [38] or mCherry [29] 

Comparing our results with the published data we conclude that N-terminal tagging of Kv1.3 channels with fluorescent proteins such as eGFP, YFP, and mKate2 does not interfere with the efficient transfer of the channel of the rat, mouse or human origin to the plasma membrane, while the extent of channel retention in ER as well as its localization in Golgi apparatus can vary for the channels of different origin [26,28,35,39,40]. 

The low number of endosomes and lysosomes containing the studied Kv1.3 variants allows us to conclude that Kv1.3 channels are rarely endocytized from the plasma membrane and their recycling is small. Moreover, interaction of Kv1.3 channels with a pore blocker does not enhance endocytosis of the channels (Figure S2D–F).

In contrast to recently reported data about localization of transiently expressed rat Kv1.3 N-terminally tagged with YFP in mitochondria of HEK293 cells [41], we did not detect any presence of the human Kv1.3 channels tagged with mKate2 in mitochondria (Figure S4). Probably, their presentation in mitochondria is too low to be detected with the confocal microscopy because of a negative influence of either mKate2 or human vs. rat sequence differences on the channel traffic to mitochondria. 

### 2.3. Interactions of A-HgTx with mKate2-Tagged Kv1.3

**Figure 4:**
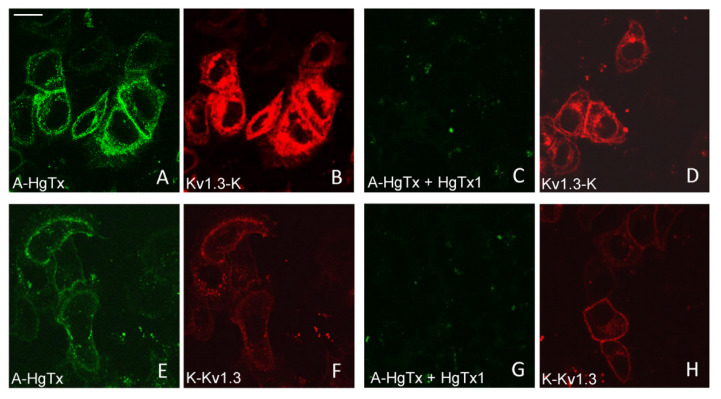

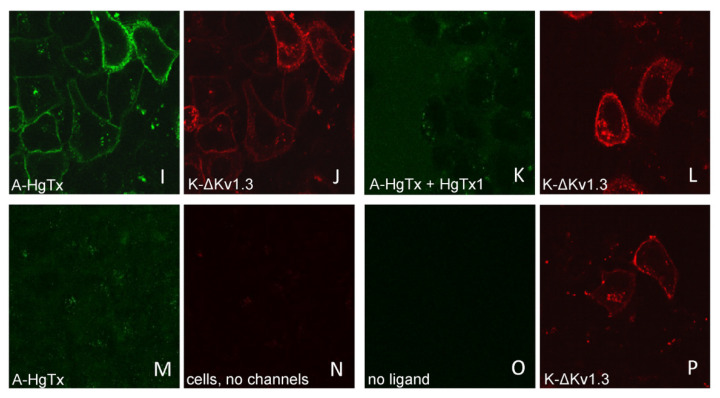
Confocal imaging of A-HgTx interactions (green) with mKate2-tagged Kv1.3 channels (red) transiently expressed in HEK293 cells. (**A**–**D**) Binding (**A**,**B**) of A-HgTx (2 nM) to Kv1.3-K and its displacement (**C**,**D**) by HgTx1 (40 nM). (**E**–**H**) Binding (**E**,**F**) of A-HgTx (2 nM) to K-Kv1.3 and its displacement (**G**,**H**) by HgTx1 (40 nM). (**I**–**L**) Binding (**I**,**J**) of A-HgTx (2 nM) to K-ΔKv1.3 and its displacement (**K**,**L**) by HgTx1 (40 nM). (**M**,**N**) Control measurement of the binding of A-HgTx (2 nM) to intact (no transfection) HEK293 cells. (**O**,**P**) Control measurement of the background signal in the wavelength range corresponding to the A-HgTx emission (**O**) from HEK293 cells expressing K-ΔKv1.3 (without addition of A-HgTx). The scale bar is equal to 20 μm. (**A**,**E**,**I,M**,**C**,**G**,**K**,**O**) Distribution of A-HgTx. (**B**,**F**,**J**,**N**,**D**,**H**,**L**,**P**) Distribution of mKate2-tagged Kv1.3 channels.

All three variants of mKate2-tagged Kv1.3 bind A-HgTx on the membrane of living HEK293 cells (Figure 4). This binding is observed at nano-molar concentrations of A-HgTx and does not lead to noticeable changes in membrane distribution of the Kv1.3 channels (Figure 1 and Figure 4). Washing cells with a fresh medium leads to rapid (in a few minutes) dissociation of A-HgTx from the cell membrane (data not shown). An excess of unlabeled HgTx1 displaces bound A-HgTx from the membrane of cells presenting any of the variants of mKate2-tagged Kv1.3 channels (Figure 4). No binding of A-HgTx was observed at the membrane of intact HEK293 cells that do not express mKate2-tagged Kv1.3 (Figure 4M,N). These data allow us to conclude that the external binding site of pore blockers is preserved in any of the variants of mKate2-tagged Kv1.3, and A-HgTx reversibly interacts with this binding site with high affinity.

Considering the aim of using cells expressing mKate2-tagged Kv1.3 for quantitative study of interactions with pore blockers, variants K-Kv1.3 and K-ΔKv1.3 look more preferable than Kv1.3-K due to the higher contrast of membrane-associated fluorescence, which facilitates image processing. In turn, we chose K-ΔKv1.3 for extended experiments, since its membrane presentation was slightly higher than that of K-Kv1.3 (Figure S1). 

### 2.4. Electrophysiological Studies of K-ΔKv1.3 and A-HgTx

The K-ΔKv1.3 channel and A-HgTx were characterized by a whole-cell patch-clamp technique. Taking into account that “the kinetic properties of the homomeric Kv channels are largely consistent across host cell lines” [42], we studied K-ΔKv1.3 presented at the membrane of mouse Neuro-2A neuroblastoma cells, which are routinely used in our electrophysiological experiments. Distribution of K-ΔKv1.3 in Neuro-2A cells was found to be similar to that in HEK293 cells (Figure S5B). 

**Figure 5:**
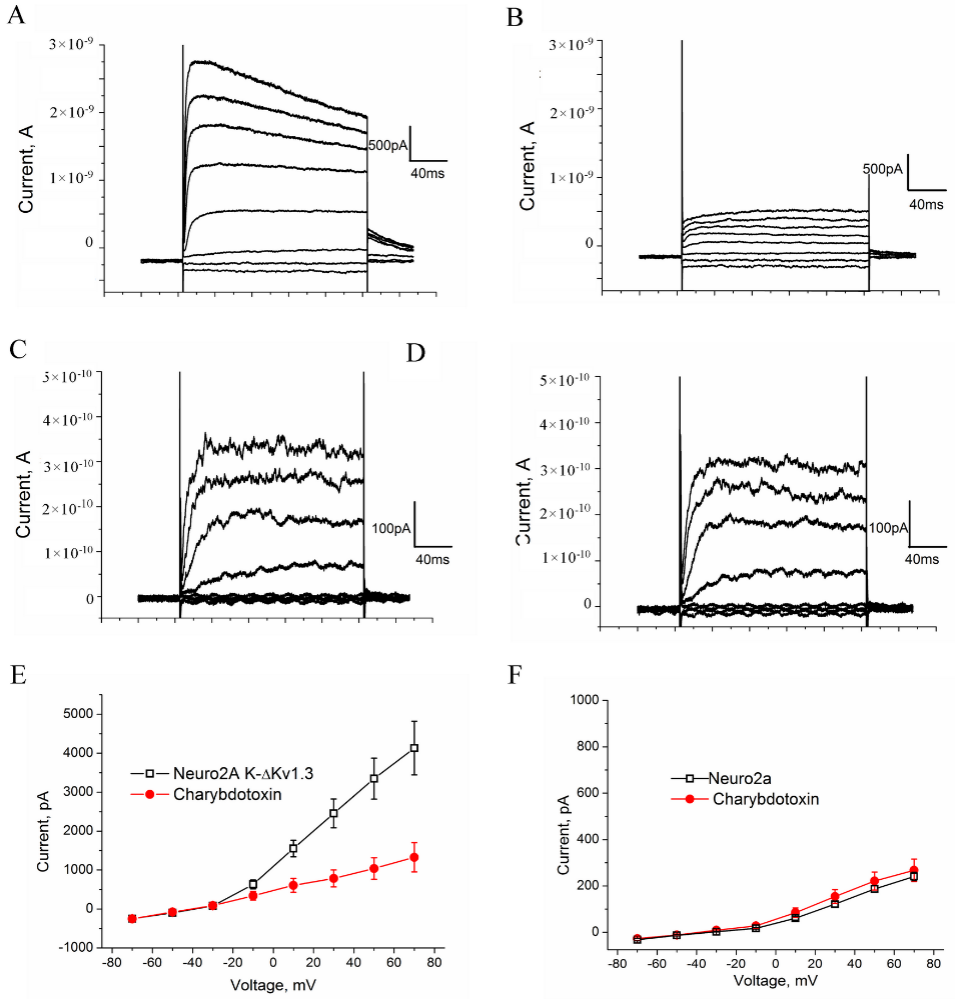
Electrophysiological characterization of K-ΔKv1.3 expressed in Neuro-2A cells. (**A**–**D**) Representative whole-cell current traces in the K-ΔKv1.3—presenting (**A**,**B**) and native control cells (**C**,**D**) in the absence (**A**,**C**) or in the presence of 20 nM ChTx (**B**,**D**) are shown. Weak currents in control Neuro-2A cells (**C**) are not affected by ChTx (**D**) in contrast to strong currents in the K-ΔKv1.3 presenting cells (**A**), which are considerably decreased after exposure of cells to ChTx (**B**). Test pulses having duration of 200 ms were applied stepwise with an interval of 20 s with a step of 20 mV from −70 to +70 mV then returning to a holding potential. (**E**,**F**) Current-voltage dependences were determined for control (**F**) and K-ΔKv1.3 presenting (**E**) cells in the absence or in the presence of 20 nM ChTx. Data were averaged (mean ± SEM) over ten (**E**) and twelve (**F**) cells. No effect of ChTx on endogenous K^+^ currents was detected in control cells.

The ion currents were analyzed in the whole-cell configuration by depolarizing steps from −70 to 70 mV in 20 mV increments from a holding potential of −40 mV. We elicited a large outward current of 2.5 to 8 nA in cells presenting K-ΔKv1.3 channels (Figure 5A). The current threshold turned out to be positive from −30 mV, and the current amplitude increased with voltage, as shown in the I-V relationships curve (Figure 5E). The application of a high-affinity Kv1.3-channel blocker ChTx [43] caused a fast inhibition of ion currents by 63 ± 4% (n = 10) at +70 mV (Figure 5B,E). 

Voltage-dependent outward ion currents were also observed in control (non-transfected) cells; however, the current threshold was positive from −10 mV, and the maximal current amplitude was in the pA range varying from 150 to 350 pA (Figure 5C,F). These currents were insensitive to the application of 20 nM ChTx (Figure 5D,F), and therefore they were not related to endogenous Kv1.3 or other ChTx binding Kv1 channels. The presence of endogenous Kv1.1, Kv1.4, Kv2.1, Kv2.1, Kv3.1, Kv4.1, Kv5.1, Kv9.2 can be supposed in Neuro-2A cells on the basis of the published data [44,45], but amplitude of their currents is low (Figure 5C,F), and these background currents do not significantly contribute to the currents measured for the cells expressing K-ΔKv1.3. 

The electrophysiology data show (Figure 5) that K-ΔKv1.3 expressed in cells is a functionally active voltage-gated channel, which is blocked by specific Kv1.3-channel blockers like ChTx. 

**Figure 6:**
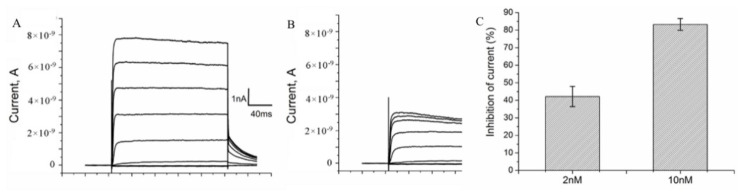
A-HgTx inhibits currents of K-ΔKv1.3 expressed in Neuro-2A cells. (**A**,**B**) Representative series of currents before (**A**) and after addition of 2 nM A-HgTx (**B**). (**C**) Inhibition of K-ΔKv1.3 currents at +50 mV by different (2 and 10 nM) concentrations of A-HgTx (mean ± SEM, n = 10 and n = 3 for 2 and 10 nM A-HgTx, respectively).

The binding of A-HgTx to K-ΔKv1.3 expressed in cells was found to be accompanied by the blocking of voltage-dependent currents (Figure 6A,B). The blocking effect of A-HgTx depends on the concentration achieving 42 ± 6 and 83 ± 3 % at 2 and 10 nM, respectively (Figure 6C). Therefore, A-HgTx binding to K-ΔKv1.3 is accompanied by the pore blocking, as in the case of unlabeled HgTx1 interacting with Kv1.3 [46]. Together with the observed displacement of A-HgTx with HgTx1 (Figure 4), this result supports the conclusion about similar spatial arrangement of A-HgTx and HgTx1 bound to the external binding site of Kv1.3 channel. This is an important property to apply A-HgTx to the study of Kv1.3 pore blockers using a competitive binding assay.

### 2.5. Quantitative Analysis of A-HgTx Binding to Cells

To quantitatively characterize formation of complexes between A-HgTx and K-ΔKv1.3 on cell membranes, the confocal images of A-HgTx bound at the membrane of the K-ΔKv1.3 presenting cells were subjected to the treatment procedure as follows. Regions of interest (ROIs) corresponding to the plasma membranes stained with K-ΔKv1.3 were selected. Using a set of these ROIs and a threshold procedure (to null a background signal from pixels without K-ΔKv1.3 signal), the binary (0;1) mask that describes membranous localization of K-ΔKv1.3 was created. The images of K-ΔKv1.3 and A-HgTx distribution were multiplied by the corresponding mask. Next, average intensities of fluorescence of K-ΔKv1.3 (*I_ci_*) and A-HgTx (*I_li_*) were calculated for each ROI (i.e., for each i-cell), and a ratio R*_i_* was calculated as follows.

*R_i_* = (*I_li_* − *I_lb_*)/(*I_ci_* − *I_cb_*) (1)

where *I_lb_* and *I_cb_* are intensities of background signals in the fluorescent images of A-HgTx and Kv1.3 channels, respectively. Calculating *R_i_* (i.e., normalizing A-HgTx intensity to that of K-ΔKv1.3), a correction was introduced for variations in a level of K-ΔKv1.3 expression on the membrane of particular cells. *R_i_* values of 15–20 cells were averaged to obtain the *R_av_* value, and a standard deviation was calculated.

### 2.6. Analysis of A-HgTx Binding to Cells

**Figure 7:**
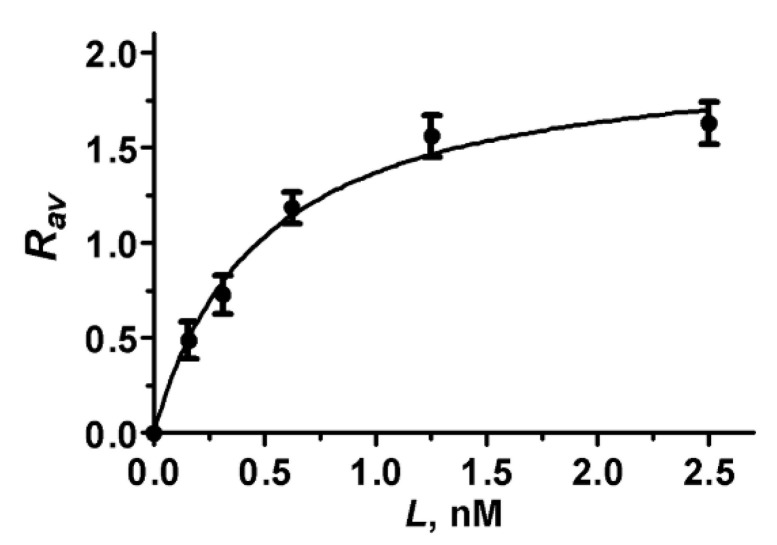
Concentration dependence of A-HgTx binding to K-ΔKv1.3 on a cell membrane that was measured as the dependence of *R_av_* on the concentration *L* of A-HgTx added to HEK293 cells.

Analysis of the *R_av_* dependence on the concentration *L* of A-HgTx added to cells (Figure 7) shows that the binding of A-HgTx to K-ΔKv1.3 is concentration-dependent and saturable. To estimate dissociation constant (*K_d_*) of the A-HgTx complexes with K-ΔKv1.3 the dependence of *R_av_* on *L* was fitted with equation

*R_av_*(*L*) = *R_m_L*/(*K_d_* + *L*) (2)

where *R_m_* is the maximal *R_av_* value corresponding to the saturation of binding. According to the performed analysis *K_d_* of complexes between A-HgTx and K-ΔKv1.3 was estimated to be 0.48 ± 0.08 nM (mean ± SEM, n = 3). In order to verify, if a truncation of the N-terminal part of Kv1.3 affects affinity of A-HgTx to the outer binding site of pore blockers, the concentration dependent binding of A-HgTx to K-Kv1.3 was measured (Figure S6), and *K_d_* of 0.30 ± 0.13 nM (mean ± SEM, n = 3) was obtained. *K_d_* values of the complexes with K-Kv1.3 and K-ΔKv1.3 differ insignificantly (*p* = 0.08) that support the choice of K-ΔKv1.3 for the studies of pore blockers. 

### 2.7. Competitive Binding Experiments

It is known that the procedure of competitive binding of a radio-labeled pore blocker and a tested compound to ion channels can be reliably used to prove the binding ability and estimate affinity of this compound to the pore binding site of the channel [14]. Here, we address the question whether a similar procedure can be implemented using fluorescent ligand A-HgTx and whole cells presenting K-ΔKv1.3. 

**Figure 8:**
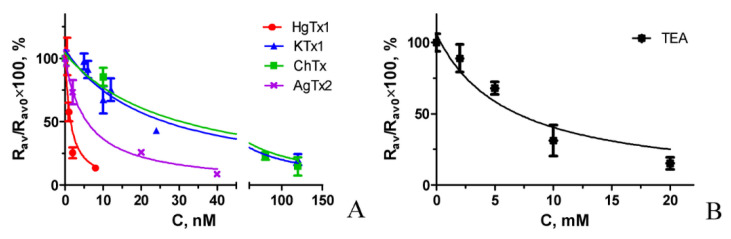
Confocal Competitive binding of A-HgTx and various pore blockers to K-ΔKv1.3 on the membrane of HEK293 cells. (**A**) Peptide pore blockers from scorpion venoms. (**B**) Non-specific pore blocker TEA. Concentration of A-HgTx was 2 nM. Concentration of pore blockers *C* varied as indicated in the graph. Representative dependences are shown for each studied pore blocker.

Competitive binding experiments revealed that A-HgTx is displaced from the complexes with K-ΔKv1.3 on the membrane of living cells by different known peptide pore blockers of Kv1.3 channel in a concentration dependent manner (Figure 8). A list of the studied peptides includes recombinant HgTx1, ChTx, AgTx2 and kaliotoxin 1 (KTx1), which were initially found in venoms of different scorpions. Moreover, A-HgTx is also displaced by the non-specific pore blocker tetraethylammonium (TEA, Figure 8), which binds to both the inner cavity of the pore [47,48] and the outer vestibule of Kv channels [49]. It has been found that the data of competitive binding experiments (Figure 8) can be approximated by the equation that is formally similar to the one used in the radioligand analysis [14]:

*R_av_*(*C*) = *R_av_*_0_/(1 + *C*/*IC*_50_) (3)

where *R_av_*(*C*) and *R_av_*_0_ are *R_av_* values at the concentration *C* of the studied compound and at *C* = 0, respectively; *IC*_50_ is the concentration of the studied compound that displaces 50% of A-HgTx from the complex with K-ΔKv1.3 channel.

When *K_d_* of A-HgTx and *IC*_50_ for the studied compound are known, the apparent dissociation constant *K_ap_* of complexes between the compound and channel can be calculated using the Cheng-Prusoff equation [50]:

*K_ap_* = *IC*_50_/(1 + *L*/*K_d_*) (4)

where *L* is the concentration of A-HgTx in the competitive binding experiment. 

The *K_ap_* values obtained for the studied pore blockers (Figure 8) were averaged over three independent measurements (mean ± SEM) and presented in Table 1. 

These data demonstrate that a combination of A-HgTx, K-ΔKv1.3 expressed on the membrane of mammalian cells, laser scanning confocal microscopy and the proposed procedure of the analysis of confocal images represent a fluorescent competitive binding assay (FCBA) that is reliable for estimation of affinities of Kv1.3 pore blockers, which interact with the outer vestibule of the channel pore. How do the affinity data obtained with FCBA correlate with the data of other methods?

According to the data of FCBA all the tested peptide blockers bind to Kv1.3 channel at low nano-molar or sub-nano-molar concentrations, while TEA is active in the millimolar concentration range (Table 1). Published data on the affinity of these pore blockers vary considerably, depending on the method and the type of cells used for the measurement (Table 1). Thus, activity of HgTx1 estimated with FCBA and with Rb^+^ efflux technique [46] is similar, but it is considerably lower than determined by radioligand analysis [46]. FCBA and a patch clamp analysis on mammalian cells [51,52] provided consistent estimation of KTx1 activity, while the data of patch clamp and voltage clamp measurements on oocytes show that the peptide activity is considerably higher [52,53]. Affinity of ChTx evaluated with FCBA and with a patch clamp technique on mammalian cells [51] is comparable, while the measurement with voltage clamp approach on oocytes show that ChTx affinity is essentially higher [43,53]. FCBA showed lower activity of AgTx2 as compared to a patch clamp technique on mouse channels in mammalian cells [51], a voltage clamp method on human channels in oocytes [53] and especially a voltage clamp technique on rat channels in oocytes [43]. Millimolar activity of TEA revealed with a patch clamp approach on mammalian cells [51] was confirmed using FCBA. 

The reasons for variations in the estimations of pore blocker activity obtained with different techniques include essential dependence of *K_d_* on ionic strength of the medium, probable influence of the components of a culture medium in the experiments on cells, unavoidable adsorption of the cationic pore blocker on materials of an experimental setup, and different origin (rodent or human) of Kv1.3 channels. Although Kv1.3 channels of different species are highly homologous proteins, some differences exist in the P-loop, which comprises the extracellular binding site for peptide blockers. Given the multi contact nature of interactions of peptide blockers with the P-loop of the target Kv1 channels [54,55], structural variations in the P-loop could affect the *K_d_* value. 

The results of our experiments demonstrate that with due attention to the factors affecting the interaction of pore blockers with the channel, the developed analytical approach based on the confocal fluorescent microscopy of the A-HgTx complexes with K-ΔKv1.3 channels presented at the membrane of living cells allows one to screen Kv1.3 blockers, which bind to the extracellular vestibule of the K^+^-conducting pore, and analyze their affinity. The presented FCBA uses a formalism of radioligand analysis, but overcomes the safety restrictions that arise when manipulating with radioactive compounds, eliminates the stages of fragmentation of channel-containing membranes, and their washing from an unbound radioactive ligand. The assay is realized in the mix-and-measure format that simplifies its application and increases reliability. 

## 3. Conclusions

Heterologous expression of Kv1.3 channels fused N- or C-terminally with a fluorescent protein is a convenient approach to the study of different aspects of biogenesis, functioning, and interactions of Kv1.3 channel in mammalian cells [25,26,27,28,29]. Tagging of Kv1.3 with the bright red fluorescent protein mKate2 extends a color variability of fully functional fluorescent Kv1.3 channels with enhanced membrane localization in mammalian cells, and expands the capabilities of multi-color fluorescent analysis. 

In the study of Kv1.3 channel and its blockers, rat or mouse *KCNA3* genes are predominantly used, as they provide robust membrane localization of the target channel [26,28,39,56,57]. In spite of the high homology between human, rat, and mouse Kv1.3 channels, clinic-oriented studies must be performed on the human channel, and membrane targeted variants of mKate2-tagged human Kv1.3 meet this requirement. 

The studies of channel blockers using FCBA are performed on cells in the conditions when the influence of non-specific interactions with cellular membrane and interference with the components of cell medium on the effective channel blocking concentration of the studied compound can be revealed. The presented approach adds to the arsenal of techniques in the research field and has the potential to assist in expansion of the library of peptide blockers of Kv1.3 channel, which are suitable for drug development. 

We believe that varying the fluorescent probe and the type of expressed channel the developed approach can be extended to the study of interactions between various groups of peptide blockers and ion channels on the cellular level. 

## 4. Materials and Methods

### 4.1. Reagents

HgTx1 N-terminally labeled with Atto488 (A-HgTx) was produced by chemical synthesis by Smartox Biotechnology (France), purity 98%. Concentration of A-HgTx was measured using molar extinction coefficient 90,000 M^−1^cm^−1^ at 500 nm. Oligonucleotide primers listed in Table 2 were synthesized by Evrogen (Russia). LTG, ERTG, TR488, and NCer were purchased from ThermoFisher Scientific (Waltham, MA, USA). Rh123, tetraethylammonium chloride (TEA) and bovine serum albumin (BSA) were from Merck (Darmstadt, Germany). GenJector-U Transfection Reagent was from Molecta (Moscow, Russia).

### 4.2. Design of Expression Plasmids Encoding of Kv1.3 Fused with mKate2

*KCNA3* gene encoding human Kv1.3 channel (hKv1.3, Accession number NP_002223.3) was a generous gift from Dr. A. Vassilevski. To construct a plasmid pmKate2-KCNA3, which encode hKv1.3 N-terminally tagged with mKate2 (K-Kv1.3) the *KCNA3* gene was amplified in PCR using oligonucleotide primers Kcna3-f1 and Kcna3-r1 (Table 1), and the resulting DNA fragment was cloned into BglII/HindIII sites of pmKate2-C expression vector (Evrogen, Russia). Plasmid pmKate2-KCNA3-del, a derivative of pmKate2-KCNA3, was obtained, as described earlier [23]. This plasmid encoded N-terminally truncated Kv1.3 (deletion M1-D52) fused at its N-terminus with mKate2 (K-ΔKv1.3).

To construct a plasmid pVax1-KCNA3-mKate2, which encoded hKv1.3 C-terminally tagged with mKate2 (Kv1.3-K), *KCNA3* gene was amplified in PCR using oligonucleotide primer Kcna3-f2 containing Kozak sequence and ATG codon and primer Kcna3-r2, in which HindIII restriction site was introduced before TAA stop codon (Table 2). The obtained DNA fragment was cloned into NheI/KpnI sites of plasmid pVax1 (Invitrogen, Carlsbad, CA, USA) to get pVax1-KCNA3. Then the HindIII/EcoRI sites of pVax1-KCNA3 were used to clone a gene coding for mKate2, which was PCR-amplified from pcmKate2-C plasmid (Evrogen, Russia) using primers mKate2-f1 and mKate2-r1. The resulting recombinant gene in the plasmid pVax1-KCNA3-mKate2 encoded hKv1.3-mKate2 fusion, in which hKv1.3 and mKate2 were separated by a short linker KASGGGS. Correct cloning of DNA inserts was confirmed by sequencing (Evrogen, Russia) of both strands in the created plasmids pmKate2-KCNA3 and pVax1-KCNA3-mKate2.

### 4.3. Recombinant Peptides

Recombinant peptides HgTx1, KTx1, AgTx2, and ChTx were obtained as described earlier [58]. 

Concentrations of HgTx1, KTx1, AgTx2, and ChTx were measured with UV spectrophotometry in an aqueous solution containing 20% acetonitrile and 0.1% trifluoroacetic acid using molar extinction coefficients (at 214 nm) 54,600, 49,300, 49,200, and 282,000 M^−1^ cm^−1^, respectively. These coefficients were calculated as proposed elsewhere [59].

### 4.4. Experiments with Cells

HEK293 cells (from the Russian collection of cell cultures, the Institute of Cytology RAS, Saint Petersburg, Russia) were grown in Dulbecco’s modified Eagle’s medium DMEM/F12 (Paneco, Russia) containing 5% fetal bovine serum (FBS, HyClone, Utah, USA) and 2 mM L-glutamine (complete medium). Cells were passaged every 72 h and used for the experiments in the period from 5 to 20 passages. 

Cells were sown on round cover glasses placed in the wells of 24-well plates ((60 ± 10)×10^3^ cell per well), and 24 h later subjected to transfection with plasmids pmKate2-KCNA3, pmKate2-KCNA3-del or pVax1-KCNA3-mKate2. Transient transfection of cells was performed using GenJector-U reagent at nearly 50% cell confluence according to the manufacturer’s protocol. Efficiency of transfection achieved usually ca. 40% as examined in 24 h using fluorescence microscopy. 

Mouse Neuro-2A neuroblastoma cells were purchased from the Russian collection of cell cultures (Institute of Cytology, Saint Petersburg, Russia). Cells were cultured in DMEM (Paneco, Russia) supplemented with 10% fetal bovine serum (PAA Laboratories, Austria) and penicillin–streptomycin 100 U/mL. Cells were subcultured twice a week. Cells were plated at a density of 3×10^4^ cells per well on cover glasses coated with poly-D-lysine and 24 h latter subjected to the transfection with plasmid pmKate2-KCNA3-del as described above. 

For staining cellular organelles, a specific probe was added to a culture medium: LTG (50 nM) for 30 min, ERTG (1 µM) for 30 min or Rh123 (125 μg·L^−1^) for 5 min. For endosome staining cells were incubated with TR488 (30 μg/mL) for 30 min in serum free media. For staining of Golgi apparatus, cells were incubated with NCer-BSA complex for 30 min at 7 °C in Hank’s solution, washed twice and incubated in Hank’s solution for 30 min at 37 °C.

In A-HgTx binding experiments, cells were incubated with A-HgTx (0.15–4 nM) for 30 min (37 °C, 5% CO_2_) in the complete medium. In competitive binding experiments, cells were incubated with A-HgTx (2 nM) and selected pore blocker at variable concentrations (0.5–100 nM for recombinant peptides and 1–20 mM for TEA) for 30 min (37 °C, 5% CO_2_) in the complete medium. 

### 4.5. Electrophysiology Measurements

K-ΔKv1.3-channel ion currents were recorded by the patch-clamp technique using an EPC-10 amplifier (HEKA Elektronik, Germany) in a standard whole-cell configuration. Micropipettes were pulled from a borosilicate glass tube with filament (Sutter Instrument, Novato, CA, USA). The resistance of a micropipette tip was 6–8 MΩ when it was filled with the pipette solution contained (mM): 140 KCl, 6 CaCl_2_, 2 MgCl_2_, 2 MgATP, 0.4 NaGTP, 10 HEPES, 20 BAPTA/KOH (pH 7.3).

Briefly, in 24–48 h after transfection, cover glasses with attached Neuro2a cells were placed in a chamber with an extracellular solution (mM): 140 NaCl, 2.8 KCl, 2 MgCl_2_, 2 CaCl_2_, 10 HEPES and 10 glucose (pH 7.4) and perfused continuously. Selection of cells for the measurements was guided by membrane fluorescence of K-ΔKv1.3. The holding potential was set at −40 mV. Test pulses of 200 ms duration were applied stepwise with 20 s intervals; the voltage varied from −70 mV to +70 mV with a step of 20 mV and finally returned to a holding potential. 

The experiments were carried out at 20–25 °C. Cells with input resistance less than 350 MΩ were excluded from analysis.

### 4.6. Confocal Microscopy

Experiments with HEK293 cells were performed with a laser scanning confocal microscope Leica-SP2 (Leica Microsystems GmbH, Wetzlar, Germany). The water-immersion 63×/1.2 NA HCX PL APO objective was used. 

Fluorescence of mKate2-fused hKv1.3 was excited at the 561 nm wavelength and recorded in the 650–700 nm range. Fluorescence of A-HgTx was excited at the 488 nm wavelength and recorded in the 495–535 nm range. Distributions of A-HgTx and mKate2 fluorescence in cells were imaged using sequential scanning to avoid interference of intrinsic cellular fluorescence excited at the 488 nm with the mKate2 fluorescence. 

In the co-localization experiments, the confocal images of mKate2-fused hKv1.3 and fluorescent probes for cellular organelles were recorded using a sequential scanning to avoid signal crosstalk. Fluorescence of LTG, ERTG, Rh123, TR488 or NCer was excited at the 488 nm wavelength and recorded in the 498–550 nm range. 

Lateral and axial resolutions in confocal imaging experiments were ca. 0.2 and 0.6 µm, respectively. 

The recorded images were subjected to the treatment with the ImageJ software (National Institutes of Health, Bethesda, MD, USA) as described in the Results section. 

Each experimental point in the curves describing interaction A-HgTx with mKate2-tagged Kv1.3 channels is an average over 15–25 treated images of cells. All the measurements were repeated in three independent experiments. 

## Figures and Tables

**Table 1:** The *K_ap_* values for the complexes formed by K-ΔKv1.3 with the studied pore blockers and published data on the affinities of these blockers.

	HgTx1	KTx1	ChTx	AgTx2	TEA
K_ap_, nM	0.2 ± 0.1	4 ± 2	7 ± 4	1.1 ± 0.3	(2 ± 1) × 10^6^
K_d_, nM	0.086 ^a^; (2.4 × 10^−4^) ^b^	0.65 ^c^; 0.1 ^d^; 2 ^e^; 0.01 ^f^	3 ^c^; 0.19 ^g^; 0.9 ^f^	0.004 ^g^; 0.05 ^f^; 0.2 ^c^	(1 × 10^7^) ^c^

^a^ Rb^+^ efflux from HEK293 cells [46]; ^b^ radioligand analysis, membranes of HEK293 cells [46]; ^c^ whole-cell patch-clamp; mouse channels, L929 mouse fibroblast cells [51]; ^d^ patch clamp, oocytes [52]; ^e^ patch clamp, Jurkat cells [52]; ^f^ two-electrode voltage clamp, human channels, oocytes [53]; ^g^ two-electrode voltage clamp, rat channels, oocytes [43].

**Table 2:** Oligonucleotide primers used in this study.

Notation	Nucleotide Sequence *
Kcna3-f1	5′-TTCTCAGATCT**ATG**GACGAGCGCCTCAGCCTTCTG-3′
Kcna3-r1	5′-TTCTCAAGC**TT****A**AACATCGGTGAATATCTTTTTGATGTTGA-3′
Kcna3-f2	5′-AAGCTGGCTAGC*GCCACC***ATG**GACGAGCGCCTCAGCCTTCTG
Kcna3-r2	5′-TTCTTCGGTACC**TTA**AGCTTTAACATCGGTGAATATCTTTTTGATGTT
mKate2-f1	5′-TTCTTCAAGCTTCTGGTGGAGGTAGC**GTG**AGCGAGCTGATTAAGGAGAAC
mKate2-r1	5′-TTCTTCGAATTC**TCA**TCTGTGCCCCAGTTTGCTAGGGA

* Restriction enzyme sites used for cloning are underlined. Start codon (ATG) in forward primers and stop codons (TAA and TGA) in reverse primers (TTA and TCA, respectively) are marked in bold. GTG codon encoding N-terminal residue of mKate2 is also marked in bold.

## Data Availability

The data presented in this study are available on request from the corresponding author. The data are not publicly available due to local regulations.

## Supplementary Materials

The following supporting information can be downloaded at: https://www.mdpi.com/article/10.3390/toxins14120858/s1, Figure S1: Comparison of fluorescence intensities of K-Kv1.3 and K-ΔKv1.3 expressed in membrane of HEK293 cells, Figure S2: Distribution of mKate2-tagged Kv1.3 (red) and the endosome marker TR488 (green) in living HEK293 cells, Figure S3: Distribution of mKate2-tagged Kv1.3 (red) and the lysosome marker LTG (green) in living HEK293 cells, Figure S4. Distribution of mKate2-tagged Kv1.3 (red) and the mitochondrion marker Rh123 (green) in living HEK293 cells, Figure S5: Confocal imaging of A-HgTx interactions with K-ΔKv1.3channels transiently expressed in Neuro-2A cells, Figure S6: Concentration dependence of A-HgTx binding to K-Kv1.3 on a cell membrane that was measured as the dependence of *R_av_* on the concentration [L] of A-HgTx added to HEK293 cells transiently transfected with K-Kv1.3.
