# Supplementary material for: Combining mKate2-Kv1.3 Channel and Atto488-Hongotoxin for the Studies of Peptide Pore Blockers on Living Eukaryotic Cells
Source: Toxins (Basel). 2022 Dec 5;14(12):858. doi: 10.3390/toxins14120858 (PMC9780825; doi:10.3390/toxins14120858)
Supplement: Supplementary file 1 [file toxins-14-00858-s001.zip › toxins-2038251-supplementary.pdf]

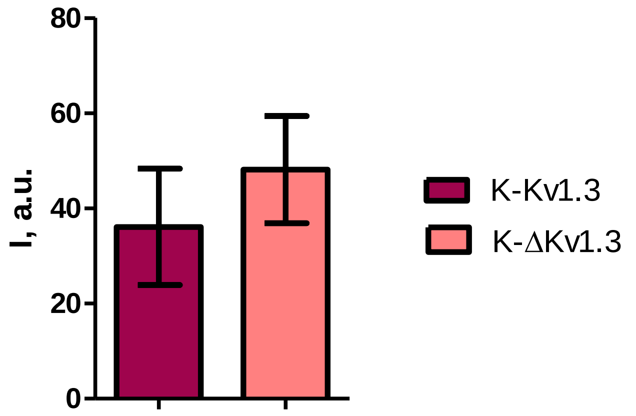

Figure S1. Comparison of fluorescence intensities of K-Kv1.3 and K-ΔKv1.3 expressed in membrane of HEK293 cells. Intensities were measured with confocal microscopy using the same parameters and treated with Image J software as described in the Results section. Data were averaged (mean ± SD, n = 20; $p = 0.47$) for each channel.

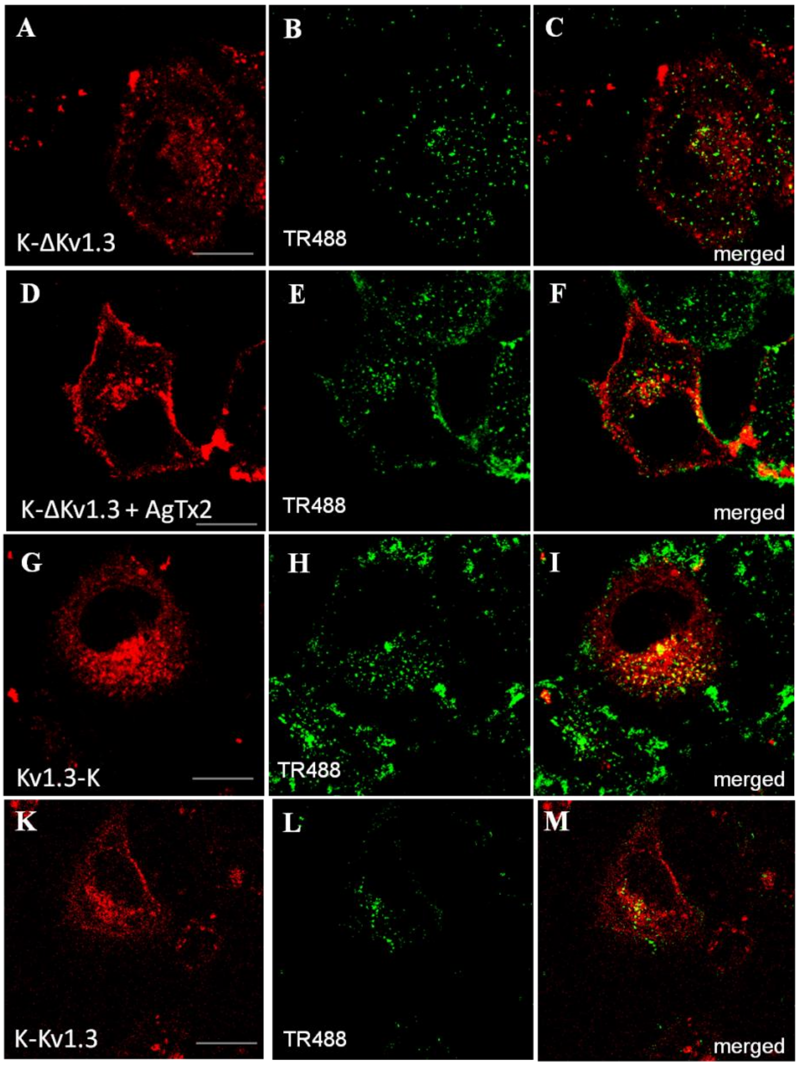

Figure S2. Distribution of mKate2-tagged Kv1.3 (red) and the endosome marker TR488 (green) in living HEK293 cells. (A-C), (D-F) Cells expressing K-ΔKv1.3. (D-F) Cells were co-incubated with agitoxin 2 (AgTx2,100 nM, 30 min) and TR488. (G-I) Cells expressing Kv1.3-K. (K-M) Cells expressing K-Kv1.3. (C,F,I,M) Merged images of mKate2-tagged Kv1.3 and TR488 fluorescence. Yellow color indicates co-localization of the channels and ERTG in endosomes. Scale bar: 20 μm.

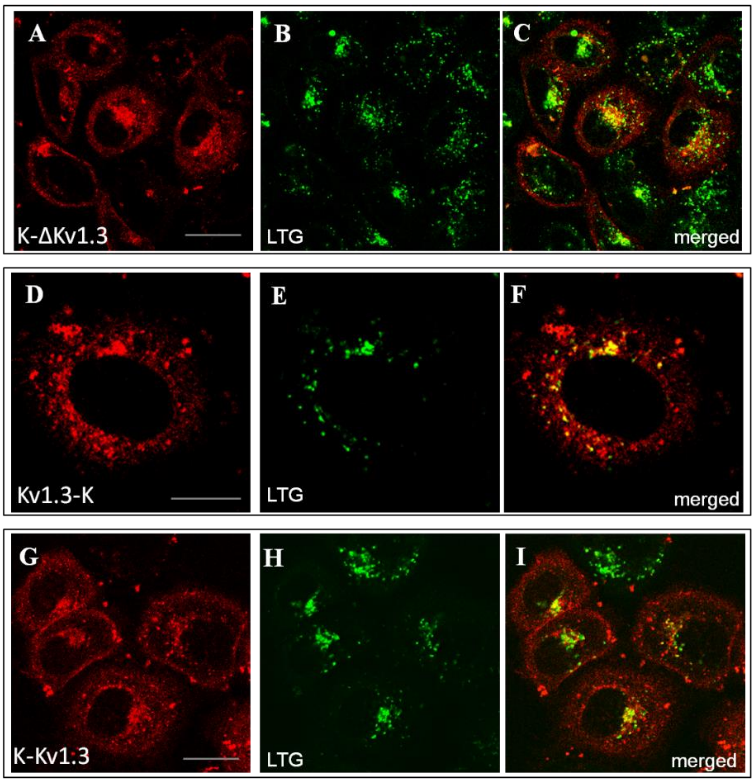

Figure S3. Distribution of mKate2-tagged Kv1.3 (red) and the lysosome marker LTG (green) in living HEK293 cells. (A-C) Cells expressing K-ΔKv1.3. (D-F) Cells expressing Kv1.3-K. (G-I) Cells expressing K-Kv1.3. (C,F,I) Merged images of mKate2-tagged Kv1.3 and LTG fluorescence. Yellow color indicates co-localization of the channels and LTG in lysosomes. Scale bar: 20 μm.

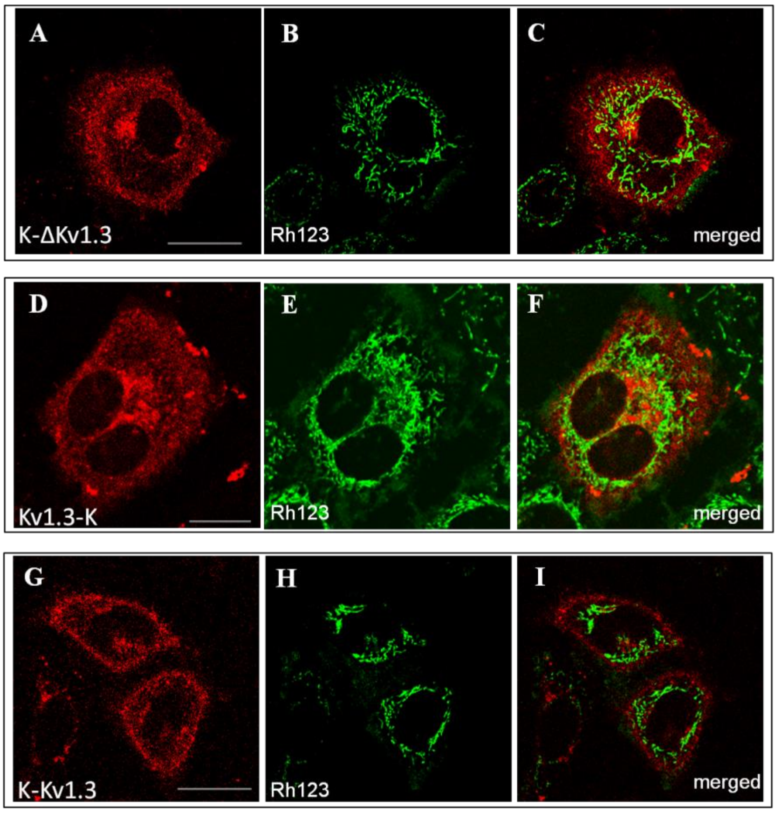

Figure S4. Distribution of mKate2-tagged Kv1.3 (red) and the mitochondrion marker Rh123 (green) in living HEK293 cells. (A-C) Cells expressing K-ΔKv1.3. (D-F) Cells expressing Kv1.3-K. (G-I) Cells expressing K-Kv1.3. (C,F,I) Merged images of mKate2-tagged Kv1.3 and Rh123 fluorescence. Scale bar is 20 μm.

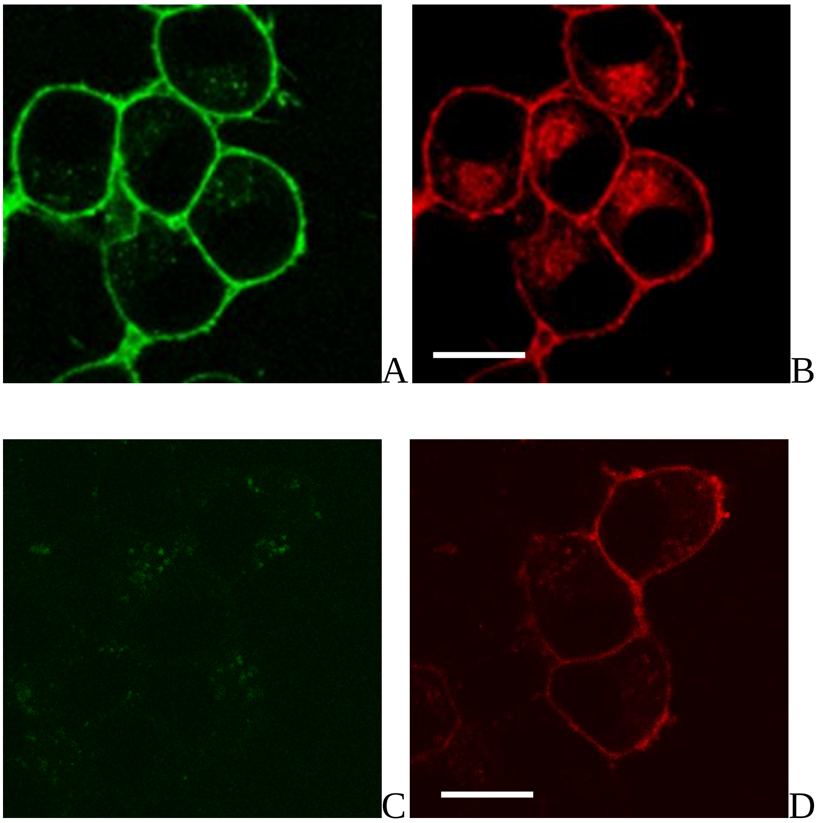

Figure S5. Confocal imaging of A-HgTx interactions with K-ΔKv1.3channels transiently expressed in Neuro-2A cells. (A, B) Distribution of A-HgTx (2 nM) bound at the membrane of cells expressing K-ΔKv1.3. (C,D) Displacement of A-HgTx (2 nM) with HgTx (20 nM) from the complexes with K-ΔKv1.3 at the membrane of cells. Scale bar is 20 μm. Green and red colors – fluorescence of A-HgTx and K-ΔKv1.3, respectively.

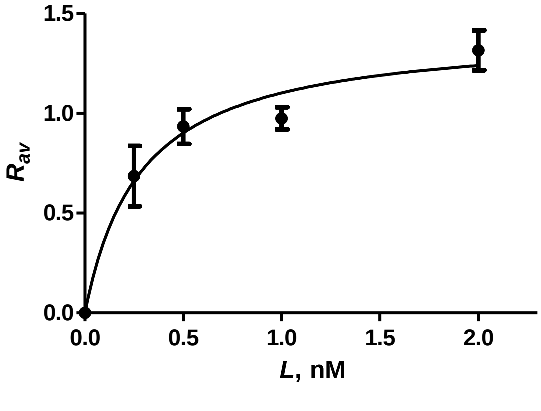

Figure S6. Concentration dependence of A-HgTx binding to K-Kv1.3 on a cell membrane that was measured as the dependence of Rav on the concentration L of A-HgTx added to HEK293 cells transiently transfected with K-Kv1.3.
